# Developing a prehospital care service in a low‐resource setting: Barriers and solutions

**DOI:** 10.1002/hsr2.1719

**Published:** 2023-11-16

**Authors:** Berjo D. Takoutsing, Yvan Zolo

**Affiliations:** ^1^ Research Department Association for Future African Neurosurgeons Yaounde Cameroon; ^2^ Research Division Winners Foundation Yaounde Cameroon; ^3^ Global Surgery Division University of Cape Town Cape Town South Africa

**Keywords:** ambulances, developing countries, emergency medical services, first aid, prehospital care

## Abstract

Prehospital care (PHC) is critical to the comprehensive and effective functioning of a healthcare system. Given the disproportionate burden of both communicable and non‐communicable diseases in low‐income nations, its significance cannot be understated. In spite of this, many of these nations lack a comprehensive PHC system. Setting up a cost‐effective PHC system in this environment can be difficult and necessitate a variety of stakeholders at various healthcare delivery system levels. Therefore, it is necessary to consider these anticipated barriers and identify feasible solutions for its execution. This will assist in creating a PHC system that is suited to the local needs and achieve sustainable and global health goals. This paper describes the challenges and solutions to establishing a prehospital care service in a low‐resource setting.

## 1 INTRODUCTION AND INSIGHTS OF PREHOSPITAL CARE SYSTEM IN RESOURCE LIMITED SETTINGS

**Figure 1:**
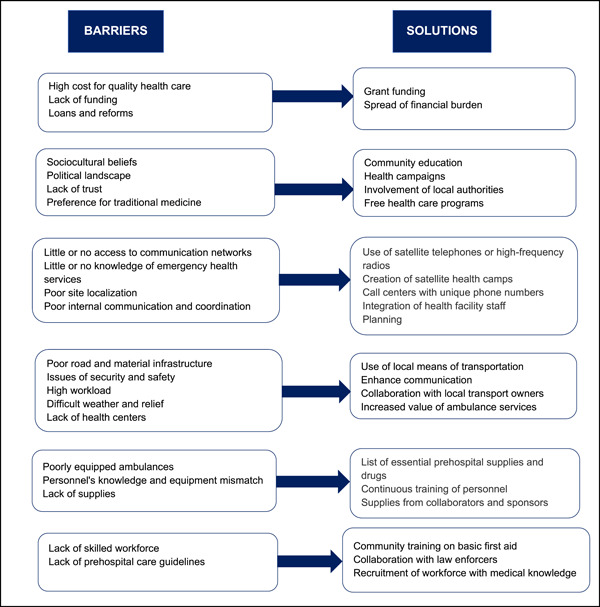
Barriers and solutions to establishing a prehospital care system in a low resource setting.

Prehospital care (PHC) is the “provision of emergency medical services (EMS) for resuscitative, preventative, analytic, and stabilizing purposes at the scene or while transporting the sick or injured to a hospital or other emergency medical facility.”^1^ When the PHC system is set up and put into practice properly utilizing suitable healthcare policies and processes, it can help decrease morbidity, mortality, and permanent disability from serious illness—lowering the burden of disease and lengthening life expectancies.^2^ This is crucial in low‐income countries (LICs), where EMS are difficult or impossible and where about 80% of deaths following severe injury occur in the prehospital setting.^1^ The development and implementation of a successful PHC system are fraught with difficulties (Figure 1), particularly in environments with limited resources.^3^ However, establishing a cost‐effective PHC system in resource‐constrained settings necessitates identifying the obstacles to its implementation as well as potential solutions (Figure 1).

## 2 THE ROLE OF FUNDING, POLITICAL, AND SOCIOCULTURAL LANDSCAPE IN ESTABLISHING PREHOSPITAL CARE SYSTEMS

Financial imbalances in a healthcare system are known to impair its smooth functioning.^4^ LICs, which rely primarily on out‐of‐pocket spending, and have few insurance plans, are likely to have a hard time affording PHC services.^5^ Due to its high cost, PHC systems are often supported by loans and reforms.^5^ These funding mechanisms are inconsistent, and unsustainable.^1^ In addition, the idea that high‐quality care is expensive may discourage the general populace from using PHC services.^5^ Applying for grant funding from the government and humanitarian organizations with common health goals are viable solutions to this issue. Similarly, spreading the financial load throughout the entire community through donations, taxation, and out‐of‐pocket funding can better the financial burden of establishing a cost‐effective PHC service.^1^

Cultural differences affect how different populations behave when seeking medical care.^6^ In rural areas, with high illiteracy, poverty, and gender bias rates, the choice to seek medical assistance rests with family members and higher authorities (chiefs, community leaders, council heads, administrators).^6^ Additionally, the preference for traditional treatments over contemporary medicine depends on widespread community approval—which is usually inclined toward traditional practices in LICs.^6^ As a result, knowledge of the danger signs of serious illnesses, the need to seek immediate care, and contact with the emergency service are low.^7^ The success of the Christian religion's growth throughout Africa serves as concrete evidence that community education can be used to remove obstacles to community access in low‐resource environments.^6^ Mindful of the cultural barriers, the education of residents in partnership with missionaries, local citizens, and traditional healthcare providers is beneficial.^6^ Also, increasing access to health campaign programs, encouraging community members to attend education sessions, and routine and preventative care at the local health facilities are important to promote knowledge and access to PHC services.^8^

## 3 COMMUNICATION AND GOVERNANCE HAS A FUNDAMENTAL ROLE IN THE EFFECTIVENESS OF PREHOSPITAL CARE SYSTEMS

There are places in LICs without access to communication networks. Additionally, there is a dearth of knowledge of an emergency number in locations where telephone lines are present.^1^ Moreover, Locating the site where PHC is needed usually presents a challenge, leading to a lack of trust from the general public in the PHC services.^1^ It will be easier to increase the prompt beginning of care by setting up a call center with a unique number(s) known by the target population that effectively communicates information to the ambulance crew directly or indirectly. The use of satellite telephones or high‐frequency radios for communication can be cost‐effective in locations lacking access to high‐tech communication resources used in high‐income countries.^9^ The administration and coordination of EMS impact the delivery of PHC.^10^ When done wrong it can lead to; a lack of appropriate triage by the dispatch team, ineffective communication among rescue team members, a lack of timely access to resources and services, and ineffective information relaid to the receiving hospital.^10^ The establishment of satellite health camps, dispatch centers, and coordination offices can bridge the lack of coordination. Also, the integration of health facility staff, community members, local government administration, and transportation authorities to act as the pillar of the emergency response system is genuine.^9^

## 4 OPTIMIZING SERVICE DELIVERY AND INFRASTRUCTURE FOR THE PROPER FUNCTIONING OF PREHOSPITAL CARE SYSTEMS

Ambulance bus services are the most popular mode of transportation for PHC providers.^10^ Nevertheless, their use in LIC is constrained by several factors impeding the timely delivery of care.^10^ These include; poor road conditions, insecurity, a lack of maintenance, an imbalance between the number of casualties and ambulances available, long travel distances, difficult relief, and harsh weather conditions.^10^ Issues with security and safety may prevent emergency responders from reaching the homes of patients in need, which are frequently underserved. This may also prevent patients from seeking care at the closest emergency care facility available in areas with inaccessible power supplies or hostile environments.^10^ For places with rough roads, the use of ambulance cars, buses, motorbikes, bicycles, and animal (horse or donkey) carts may be a more affordable option.^11^ These alternate modes of transportation ought to act as quick, intermediary methods of getting people from difficult‐to‐reach places to dispatch centers, where an ambulance with the appropriate equipment can take over. The use of cutting‐edge techniques, such as mobile phones or local communication tools,^10^ will aid in the coordination of basic first aid affected by the population, and the readiness of the healthcare facility to improve timely care. The population can be made more aware of the value of ambulance services—which will lessen traffic delays brought on by other drivers. Also, awareness of the services provided by various nearby healthcare institutions will boost the desire of local transport owners to volunteer to carry patients to the nearest health center or dispatch center with the required level of care.

Despite the availability of ambulance vehicles in low‐resource settings, many are branded buses with rudimentary or no basic functional equipment.^12^ This prevents PHC providers from providing tailored care while transporting patients to the hospital.^12^ Additionally, there is a mismatch between the personnel's knowledge and the equipment when it is available.^12^ Moreover, health centers are frequently absent, and the few ones that do exist are overcrowded and lack the required infrastructure to provide patients with the level of care they expect.^10^ In light of this, a list of essential prehospital supplies and drugs should be developed based on the most prevalent emergency health conditions which exist locally. The personnel responsible for PHC should have adequate knowledge of the usage of this equipment.^12^ To avoid situations of exhausted equipment, methods of acquiring and replenishing the equipment such as assistance from mission campaigns or non‐governmental organizations, supplies from partner hospitals, and funding from the government and populations are important.^10^

## 5 LIMITATIONS AND SOLUTIONS TO SCALEUP SKILLED WORKFORCE FOR PREHOSPITAL CARE SERVICES

The workforce of the PHC service is important for its proper running. The lack of a skilled workforce, and the lack of PHC guidelines in the health system—leave PHC providers incompetent to identify and manage acute illnesses or injuries.^13^ This may also contribute to the adverse effects of inappropriate treatment. Additionally, non‐medical personnel are known to take over the response duties at the scene of an injury usually with no prior training in first aid.^14^ Mindful of all these facts, it will be a good initiative to adequately train the entire population in basic first aid for the most frequently encountered illnesses or injuries in the community.^14^ Also, it will be a good initiative to teach police officers and firefighters the fundamentals of first aid so that they can complement the PHC team, particularly in places with a shortage of staff.^15^ Additionally, structured guidelines or standards and regulations on PHC need to be established by the health systems of LICs.^13^ When recruiting PHC staff, preference should be given to unemployed health personnel to completely meet the daily call volume of emergency calls and patient transfers.^13^ Moreover, the primary focus of training and education of newly recruited PHC human resources should be tailored triage techniques based on the local difficulties in accessing care.

## 6 CONCLUSION

The barriers to establishing a PHC system in a low‐resource setting are multiple and interconnected. Finance, culture, communication, coordination, infrastructure, transport, and workforce are the most common barriers. For the PHC systems in this region to be successful, these barriers need to be addressed with the efforts of multiple stakeholders at different levels of healthcare delivery. More importantly, these measures need to be targeted, measurable, equitable, and proven to work. Overall if implemented in a strategic way, PHC systems can help attain sustainable development by reducing morbidity, mortality, and permanent disability from serious illness—lowering the burden of disease and lengthening life expectancies.

## AUTHOR CONTRIBUTIONS

**Berjo D. Takoutsing**: Conceptualization; data curation; methodology; project administration; validation; writing—original draft; writing—review and editing. **Yvan Zolo**: Data curation; investigation; validation; writing—review and editing.

## CONFLICT OF INTEREST STATEMENT

The authors declare no conflict of interest.

## TRANSPARENCY STATEMENT

The lead author Berjo D. Takoutsing affirms that this manuscript is an honest, accurate, and transparent account of the study being reported; that no important aspects of the study have been omitted; and that any discrepancies from the study as planned (and, if relevant, registered) have been explained.

## Data Availability

There are no data associated to this paper.
